# Hyperandrogenism in menopause: a case report and literature review

**DOI:** 10.1186/2054-7099-1-7

**Published:** 2015-05-19

**Authors:** Andrey V Dolinko, Elizabeth S Ginsburg

**Affiliations:** Division of Reproductive Endocrinology and Infertility, Department of Obstetrics, Gynecology and Reproductive Biology, Brigham and Women’s Hospital and Harvard Medical School, Boston, MA USA

**Keywords:** Testosterone, Postmenopausal androgenization, Androgenization, Menopause, GnRH agonist

## Abstract

Hyperandrogenism is an uncommon diagnosis in postmenopausal women. In this case, we report on a 69-year-old postmenopausal woman who presented with several months of worsening hirsutism of the face, neck, and chin, which was confirmed on examination. Laboratory testing revealed markedly elevated testosterone levels and typical post-menopausal gonadotropin levels. Transvaginal ultrasonography and pelvic and abdominal magnetic resonance imaging (MRI) failed to reveal an ovarian or adrenal abnormality. The patient was a poor surgical candidate and was counseled to start on gonadotropin releasing hormone (GnRH) agonist therapy. Administration of leuprolide resulted in a dramatic decline in testosterone levels. The patient reported significant “hot flashes”, difficulty sleeping, anxiety, and depression secondary to treatment, and patient discontinued leuprolide therapy 3 months after initiation. To our knowledge, this is the first case that describes a woman being treated with a GnRH agonist for hyperandrogenism subsequently discontinuing GnRH agonist treatment due to significant side-effects. This case also highlights the difficulty of prescribing appropriate but off-label use of expensive medications not covered by insurance in a senior population of limited income.

## Background

Approximately 10% of all women present with hyperandrogenism associated with hirsutism at some stage in their life [1]. Possible endogenous sources of the elevated androgen levels include ovarian tumors [2–10], ovarian hyperthecosis (i.e. hyperplasia of androgenic ovarian tissue) [11–17], and adrenal tumors [18–21]. Besides the obvious virilizing physical effects of elevated testosterone, hyperandrogenism is also associated with hypercholesterolemia, insulin resistance, hypertension, and cardiac disease [22]. Because of these significant adverse effects on the health of postmenopausal women, the elimination of the source of elevated testosterone is essential. However, the identification of that source often poses a clinical challenge. If an ovarian or adrenal source is identified on imaging, oophorectomy or adrenalectomy, respectively, is often curative. However, if the source of elevated testosterone is not identified or surgery is not a safe option, treatment choices are limited. Successful medical management with GnRH agonist/analogues or antagonists has been reported in the literature. We report the case of postmenopausal hyperandrogenism of presumed ovarian androgen in a patient who was a poor surgical candidate, and we discuss the difficulties associated with medical management with a GnRH agonist.

## Case presentation

A 69 year-old post-menopausal woman presented to her primary care physician with several months of increasing terminal hair growth on her face, torso, and arms, requiring shaving. There was also recession of the hairline, but no clitoromegaly. An initial endocrine evaluation demonstrated a markedly elevated serum total testosterone of 160 ng/dL (normal 5-32 ng/dL). ACTH level was normal, and the DHEA-S level was below assay detection. Initial transvaginal pelvic ultrasound demonstrated two simple cysts measuring 11 × 12 × 8 mm and 11 mm, and a 6 × 6 × 7 mm hypoechoic structure in the right adnexa; the left ovary was not visualized. A follow-up transvaginal pelvic ultrasound demonstrated a normal right ovary measuring 17 × 5 mm and a 13 × 9 mm left paratubal cyst; the left ovary was again not seen. An MRI of the pelvis did not demonstrate any adnexal masses, and an adrenal MRI was unremarkable as well.

Her past obstetrical history was notable for six pregnancies, one vaginal delivery, one tubal ectopic pregnancy, and four 2^nd^ trimester spontaneous abortions. Her past medical history included Crohn’s disease status-post total colectomy and ileostomy. Her condition was complicated further by fistula formation through an appendectomy scar, stoma relocation, hernia repair, and development of a secondary large parastomal hernia. These operations resulted in scarring of the inside of her entire abdomen and replacement of her left lower quadrant by the large parastomal hernia. She also had chronic renal insufficiency with the serum creatinine elevated to 1.3-1.6 mg/dL. Other less significant factors in her medical history were renal stones status-post surgical removal, polymyalgia rheumatica, restless leg syndrome, asthma, arthritis, and osteoporosis, and depression.

On physical examination, the patient’s height was 63 in and weight was 157 lb (body mass index, 27.9 kg/m^2^). Her blood pressure was 122/81 mmHg. Skin examination confirmed the presence of extensive hirsutism of the face, neck and chin, and significant frontotemporal balding.

Her medications at initial evaluation included an albuterol inhaler, fluticasone, benzonatate, lactobacillus, calcium carbonate, cholecalciferol, cyanocobalamin, fenofibrate, omeprazole, zolpidem, citalopram, gabapentin, hydrocodone, pramipexole, furosemide, and zoledronic acid, none of which are known to have androgenic side effects.

Her family history was non-contributory.

Given the low levels of DHEA-S and normal adrenal imaging, an adrenal source for the testosterone was thought to be unlikely, and an ovarian source was suspected. The patient was referred to a gynecologic oncologist for possible surgical exploration and oophorectomy; however, she was deemed to be a poor operative candidate due to her multiple prior abdominal surgeries and massive lower left quadrant parastomal hernia. The patient was then referred to the reproductive endocrinology clinic for possible medical management.

Physical examination confirmed a cheerful, elderly white female with an evident shaven beard, and hairline recession. Laboratory testing demonstrated: testosterone 100.6 ng/dL (normal 2.9-40.8 ng/dL), FSH 112.1 mIU/mL (normal 25.8-134.8 mIU/mL), LH 62.4 mIU/mL (normal 7.7-58.5 mIU/mL), and estradiol 9 pg/mL (normal 0-47 pg/mL). Her hemoglobin A1c at this time was 5.9% (normal <6.0%). Lipid levels several months prior to presentation were: cholesterol 142 mg/dL (normal <200 mg/dL), HDL 51 mg/dL (normal >40 mg/dL), triglycerides 101 mg/dL (normal <150 mg/dL), and a calculated LDL 71 mg/dL (normal <129 mg/dL). A recommendation was given to start a GnRH agonist (leuprolide) to determine whether her testosterone production was gonadotropin-responsive. However, the patient did not start the GnRH agonist at that time for two reasons. First, the testosterone level had decreased somewhat on its own, though it was still quite elevated (160 ng/dL to 100 ng/dL). Second, the patient’s health insurance, Medicare, would not cover the off-label use of the medication, and the patient could not afford an out-of-pocket cost of ~ $1000/month for the intramuscular depot injection of leuprolide.

Over the next two years, the patient had a progression of her symptoms, including continued scalp hair loss requiring a wig, increased body and facial hair requiring daily shaving, increased body odor and libido, and a deepening of the voice. Several high-resolution transvaginal ultrasounds over these two years continued to demonstrate a grossly normal right ovary (1.7 cm × 0.5 cm) The left ovary was visualized on the most recent ultrasound, and was also grossly normal (1.7 cm × 0.5 cm). Total testosterone levels remained elevated up to 151 ng/dL (normal 8-60 ng/dL). Because the patient continued to be a high risk surgical candidate with no clear surgical target, the patient was referred back to the reproductive endocrinology clinic to reconsider medical management.

At the time of that appointment, her testosterone had increased to 187.4 ng/dL (normal 2.9-40.8 ng/dL) and free testosterone was 1.3 pg/mL (normal 0.14-1.72 pg/mL). The patient was started on 20 U (1 mg) of daily subcutaneous injection of leuprolide (~$300/month), which her husband helped her administer at home. Two weeks later, testosterone levels had normalized to 23.9 ng/dL, confirming gonadotropin-responsive testosterone secretion, most likely of ovarian etiology.

Unfortunately, two months after starting leuprolide, the patient reported significant “hot flashes”, difficulty sleeping, and feeling anxious and depressed. On laboratory evaluation, the total and free testosterone levels were suppressed to below assay range <2.5 ng/dL (normal 2.9-40.8 ng/dL) and <0.43 pg/mL (normal 0.14-1.72 pg/mL), respectively. The patient was advised to use half of an estradiol/norethindrone acetate patch twice weekly to relieve symptoms. However, her depressive symptoms and fatigue persisted even in the presence of add-back therapy, and given the high cost of the medication not covered by Medicare, the patient decided against pursuing further attempts at increasing progestin add-back therapy and discontinued the leuprolide one month later (three months after starting injections). At follow-up 2 weeks after discontinuing leuprolide, the patient’s testosterone had increased slightly to 6.5 ng/dL (normal 2.9-40.8 ng/dL).

Her lipid levels at this time were as follows: cholesterol 149 mg/dL (normal 100-199 mg/dL), HDL 39 mg/dL (normal 40-80 mg/dL), triglycerides 280 mg/dL (normal 35-150 mg/dL), and a calculated LDL 54 mg/dL (normal 50-129 mg/dL). The patient’s hemoglobin A1c level was 6.4% (normal <6.0%); with the aim of lowering her glucose levels and potentially ameliorating the hirsutism, she was subsequently started on metformin. At the last check-in, six months after discontinuing leuprolide therapy, her testosterone level increased to 85.24 ng/dL (normal 14-76 ng/dL), and she reported still having to shave her chin, but not as frequently as in the past. She reported being generally in good spirits, although low on energy secondary to having been recently hospitalized for medical complications unrelated to the elevated testosterone.

## Conclusions

Approximately 10% of all women present with hyperandrogenism associated with hirsutism at some stage in their life [1]. Between 1 in 300 and 1 in 1000 patients who present with hirsutism will be diagnosed with an ovarian androgen secreting tumor, which in turn account for <0.5% of all ovarian tumors [1, 10]. These rare tumors include histologies such as Leydig cell [2, 3, 5, 7, 8, 10], Brenner [4, 9], and granulosa cell tumors [6]. In addition to tumorous sources, which often present acutely, hyperandrogenism can be the result of several non-tumorous ovarian pathologies, which are more likely to present with progressive signs and symptoms of elevated androgen levels. Ovarian hyperthecosis, which results from hyperplasia of androgenic ovarian tissue, is most often diagnosed in premenopausal women under the age of 35 years; however, postmenopausal women with ovarian hyperthecosis-induced virilization have been reported in the literature as well [11–17]. Another non-tumorous source of ovarian hyperandrogenism is polycystic ovary syndrome (PCOS). While PCOS is the most common cause of hyperandrogenism in pre-menopausal women and its signs and symptoms typically ameliorate during perimenopause, it can be considered in the differential diagnosis of slowly-progressing post-menopausal hirsutism if the patient already carries a diagnosis of PCOS [23].

In addition to the ovary, the adrenal gland is another major source of androgens in postmenopausal women. Thus it is not surprising that both tumorous and non-tumorous adrenal sources of hyperandrogenism have been identified. Adrenal tumors, including non-malignant adenomas and malignant carcinomas, have been implicated in postmenopausal virilization, though they are less common than ovarian sources [18–21]. Non-tumorous adrenal sources include congenital adrenal hyperplasia, which is typically diagnosed at an early age but may have a worsening of hyperandrogenism after menopause, as well as Cushing’s syndrome and acromegaly [23]. Finally, it is critical to not miss iatrogenic causes of hyperandrogenism or the oft-elusive self-administration of androgenic drugs, including androgens, anabolic steroids, and anti-epileptic medications [23].

Besides the obvious virilizing physical effects of elevated testosterone, hyperandrogenism has other adverse effects that warrant treatment. High androgens lead to decreased HDL and increased LDL and triglyceride levels. Elevated testosterone levels are further associated with increased insulin resistance, worse hypertension, and fluid retention. Cardiac disease risk subsequently increases, bringing with it associated morbidity and mortality [22].

However, the identification of the source of elevated testosterone levels often poses a clinical challenge, and diagnostic plans typically proceed along several routes. Biochemical testing aims to differentiate between adrenal and ovarian sources. Isolated elevated testosterone, as in this case, is typically presumed to be of ovarian origin, although it has also been reported in the case of adrenal tumors [18, 19, 21, 24, 25]. Imaging is thus appropriately directed towards the ovaries and adrenals. In ovarian hyperthecosis, unilateral or bilateral ovarian enlargement may be noted on ultrasound or MRI. Adrenal or ovarian tumors may be identified by ultrasound, MRI, or CT. However, imaging is often non-diagnostic; for example, many Leydig cell tumors are less than 1 cm in size and escape detection, even on high-resolution transvaginal ultrasound [26]. In addition to imaging, the low-dose dexamethasone suppression test has been used to attempt to differentiate tumorous from non-tumorous sources of androgens of ovarian or adrenal origin. One small study showed 100% sensitivity and 88% specificity, with administration of dexamethasone failing to suppress elevated levels of testosterone, androstenedione, and DHEA-S in patients with tumorous sources [27]. Another study resulted in the development of an algorithm that suggested that lack of suppression of DHEA-S in response to low-dose dexamethasone should lead to evaluation for the presence of an adrenal tumor, while lack of suppression of testosterone could be due to either an adrenal or an ovarian tumor [28]. In cases when surgery is a safe treatment option, confirmatory testing can be carried out via ovarian and/or adrenal venous sampling to measure local testosterone levels and target the source of abnormality.

If the precise source of the excess testosterone is identified to be of ovarian or adrenal origin, oophorectomy or adrenalectomy, respectively, is typically curative. In these cases, final pathology results provide a definitive diagnosis. In instances of advanced adrenocortical carcinoma, mitotane and chemotherapy may be necessary as adjuvant therapy [23]. However, if the source of elevated testosterone is not identified or surgery is not a safe option, as in the case of this patient, treatment choices are limited.

Of note, some ovarian [2, 11, 16, 17, 26, 29–31] and adrenal [18] testosterone-producing tumors are reported to be gonadotropin-responsive. This is supported by evidence that administering a GnRH agonist or antagonist in these cases can markedly decrease testosterone levels. However, the majority of cases that have reported the use of GnRH agonists/analogues [11, 17, 30] or antagonists [2, 26, 31] used those medications as a diagnostic tool to confirm ovarian sources of excess testosterone production, rather than as long-term management. One case report described the successful use of monthly injections of leuprolide as a temporizing measure while a patient awaited surgery for 6 months after suffering a myocardial infarction [15]. A second case report described a woman who was unwilling to undergo surgery because of the risk of worsening renal failure; she was treated with monthly depot injections of leuprolide for 15 months. Notably, after discontinuing leuprolide therapy she remained free of excess testosterone production for at least 4 years of follow-up [29]. A third case described a post-menopausal woman with elevated testosterone who was a poor surgical candidate. She was treated with depot injections of leuprolide for a total of 16 months; testosterone levels dropped within 1 month of initiating the injections and remained low for 5 months after discontinuing the medication [32]. All of these patients’ clinical signs of elevated testosterone diminished as their testosterone levels decreased.

Notably, all of these cases had unconfirmed sources of the testosterone excess. Ovarian sources were suspected in all three patients, but as an adrenal gonadotropin-responsive tumor has been reported in the literature [18], those suspicions could not be confirmed.

Other possible treatment options for hyperandrogenism include the use of anti-androgens and metformin. The former include androgen receptor blockers, such as flutamide and spironolactone, as well as 5α-reductase inhibitors, such as finasteride. These have been shown to be effective in ameliorating hirsutism associated with non-tumorous causes of hyperandrogenism, including PCOS [33]. Metformin, which has been successfully used in women with PCOS in the treatment of metabolic and reproductive derangements in women of reproductive age, has not been studied in hyperandrogenic post-menopausal women, nor has it been shown to be more efficacious than placebo or anti-androgens in improving hirsutism in women with PCOS [34]. In cases of adrenal hyperandrogenism, glucocorticoid therapy may also be effective in suppressing androgens, specifically DHEA and DHEA-S, and improving symptoms and because androgens are more sensitive to glucocorticoids than cortisol, the latter should not be suppressed with administration of a low dose glucocorticoid [35].

Similar to the cases of gonadotropin-responsive tumors previously described, no source of the patient’s elevated testosterone could be confirmed through imaging, and due to her surgical history a bilateral salpingo-oophorectomy could not be safely performed. This left medical therapy as the only remaining treatment option for this patient, and leuprolide was successful in markedly decreasing the patient’s testosterone levels to normal range. While none of the cases described earlier reported on any adverse effects resulting from the leuprolide therapy, GnRH agonists are known to have multiple significant side effects, including hot flashes/sweating, edema, headache, asthenia, insomnia, and depression/emotional lability [36]. Unfortunately, the patient experienced several of these side-effects. Notably, this is the first case to our knowledge that reports a patient using GnRH agonist treatment as a means to suppress hyperandrogenism subsequently discontinuing treatment due to significant side-effects. Nevertheless, this case reinforces that a GnRH agonist should be attempted as a medical therapy in post-menopausal women who present with virilization due to elevated testosterone levels and are unable or unwilling to undergo surgical management. In addition, it highlights the difficulty of prescribing appropriate but off-label use of expensive medications in a senior population of limited income.

## Consent

Case reports are exempt per the Partners HealthCare IRB.

## Authors’ information

Please address all correspondence and request for reprints to: Dr. Elizabeth S. Ginsburg, MD, Brigham and Women’s Hospital, Department of Ob/Gyn, 75 Francis Street, ASBI-3-3254, Boston, MA 02115.

## Abbreviations

GnRH: Gonadotropin releasing hormone
ACTH: Adrenocorticotropic hormone
DHEA-S: Dehydroepiandrosterone-sulfate
MRI: Magnetic resonance imaging
CT: Computed tomography
LH: Luteinizing hormone
FSH: Follicle stimulating hormone.

## References

[1] Klotz RK, Muller-Holzner E, Fessler S, Reimer DU, Zervomanolakis I, Seeber B (2010). Leydig-cell-tumor of the ovary that responded to GnRH-analogue administration - case report and review of the literature. Exp Clin Endocrinol Diabetes.

[2] Chantler DJ, Gordon D, Millan D, Panarelli M (2011). Use of cetrorelix in the investigation of testosterone excess in a postmenopausal woman. BMJ case reports.

[3] Cvijovic G, Yamashita SA, Micic D, Kendereski A, Sumarac-Dumanovic M, Zoric S (2007). Low leptin level in an obese hyperandrogenic woman–potential marker for androgen-secreting tumor. Gynecol Endocrinol.

[4] de Lima GR, de Lima OA, Baracat EC, Vasserman J, Burnier M (1989). Virilizing Brenner tumor of the ovary: case report. Obstet Gynecol.

[5] Hofland M, Cosyns S, De Sutter P, Bourgain C, Velkeniers B (2013). Leydig cell hyperplasia and Leydig cell tumour in postmenopausal women: report of two cases. Gynecol Endocrinol.

[6] Jarabak J, Talerman A (1983). Virilization due to a metastasizing granulosa cell tumor. Int J Gynecol Pathol.

[7] Kennedy L, Traub AI, Atkinson AB, Sheridan B (1987). Short term administration of gonadotropin-releasing hormone analog to a patient with a testosterone-secreting ovarian tumor. J Clin Endocrinol Metab.

[8] Nardo LG, Ray DW, Laing I, Williams C, McVey RJ, Seif MW (2005). Ovarian Leydig cell tumor in a peri-menopausal woman with severe hyperandrogenism and virilization. Gynecol Endocrinol.

[9] Takeuchi K, Kitazawa S, Wakahashi S, Sugimoto M, Morizane M, Maruo T (2006). A case of virilizing brenner tumor in a postmenopausal woman with stromal androgenic activity. Int J Gynecol Pathol.

[10] Yetkin DO, Demirsoy ET, Kadioglu P (2011). Pure leydig cell tumour of the ovary in a post-menopausal patient with severe hyperandrogenism and erythrocytosis. Gynecol Endocrinol.

[11] Barth JH, Jenkins M, Belchetz PE (1997). Ovarian hyperthecosis, diabetes and hirsuties in post-menopausal women. Clin Endocrinol (Oxf).

[12] Buhler-Christen A, Tischler V, Diener PA, Brandle M (2009). New onset alopecia and hirsutism in a postmenopausal women. Gynecol Endocrinol.

[13] Goldman JM, Kapadia LJ (1991). Virilization in a postmenopausal woman due to ovarian stromal hyperthecosis. Postgrad Med J.

[14] Kim Y, Marjoniemi VM, Diamond T, Lim A, Davis G, Murrell D (2003). Androgenetic alopecia in a postmenopausal woman as a result of ovarian hyperthecosis. Australas J Dermatol.

[15] Krug E, Berga SL (2002). Postmenopausal hyperthecosis: functional dysregulation of androgenesis in climacteric ovary. Obstet Gynecol.

[16] Lindgren R, Gunnarsson C, Jakobsson A, Hammar M (2000). Hypersecretion of ovarian androgens may be gonadotrophin dependent many years after menopause. Maturitas.

[17] Manieri C, Di Bisceglie C, Fornengo R, Grosso T, Zumpano E, Calvo F (1998). Postmenopausal virilization in a woman with gonadotropin dependent ovarian hyperthecosis. J Endocrinol Invest.

[18] Goodarzi MO, Dawson DW, Li X, Lei Z, Shintaku P, Rao CV (2003). Virilization in bilateral macronodular adrenal hyperplasia controlled by luteinizing hormone. J Clin Endocrinol Metab.

[19] Pollock WJ, McConnell CF, Hilton C, Lavine RL (1986). Virilizing Leydig cell adenoma of adrenal gland. Am J Surg Pathol.

[20] Souto SB, Baptista PV, Braga DC, Carvalho D (2014). Ovarian Leydig cell tumor in a post-menopausal patient with severe hyperandrogenism. Arq Bras Endocrinol Metabol.

[21] Werk EE, Sholiton LE, Kalejs L (1973). Testosterone-secreting adrenal adenoma under gonadotropin control. N Engl J Med.

[22] Rothman MS, Wierman ME (2011). How should postmenopausal androgen excess be evaluated?. Clin Endocrinol (Oxf).

[23] Markopoulos MC, Kassi E, Alexandraki KI, Mastorakos G, Kaltsas G (2015). MANAGEMENT OF ENDOCRINE DISEASE: Hyperandrogenism after menopause. Eur J Endocrinol.

[24] Del Gaudio AD, Del Gaudio GA (1993). Virilizing adrenocortical tumors in adult women. Report of 10 patients, 2 of whom each had a tumor secreting only testosterone. Cancer.

[25] Vasiloff J, Chideckel EW, Boyd CB, Foshag LJ (1985). Testosterone-secreting adrenal adenoma containing crystalloids characteristic of Leydig cells. Am J Med.

[26] Stephens JW, Katz JR, McDermott N, MacLean AB, Bouloux PMG (2002). An unusual steroid-producing ovarian tumour: Case report. Hum Reprod.

[27] Kaltsas GA, Isidori AM, Kola BP, Skelly RH, Chew SL, Jenkins PJ (2003). The value of the low-dose dexamethasone suppression test in the differential diagnosis of hyperandrogenism in women. J Clin Endocrinol Metab.

[28] Abraham GE, Maroulis GB, Boyers SP, Buster JE, Magyar DM, Elsner CW (1981). Dexamethasone suppression test in the management of hyperandrogenized patients. Obstet Gynecol.

[29] Barnes RB, Ehrmann DA (1997). Long-term suppression of testosterone after treatment with a gonadotropin-releasing hormone agonist in a woman with a presumed testosterone secreting ovarian tumor. J Clin Endocrinol Metab.

[30] Pascale M-M, Pugeat M, Roberts M, Rousset H, Déchaud H, Dutrleux-Berger N (1994). Androgen suppressive effect of GnRH agonist in ovarian hyperthecosis and virilizing turnours. Clin Endocrinol (Oxf).

[31] Moore EK, Padwick ML, Irvine LM (2013). Novel management for virilisation in a postmenopausal woman. BJOG.

[32] Cheng V, Doshi KB, Falcone T, Faiman C (2011). Hyperandrogenism in a postmenopausal woman: diagnostic and therapeutic challenges. Endocr Pract.

[33] Pasquali R, Gambineri A (2014). Therapy in endocrine disease: treatment of hirsutism in the polycystic ovary syndrome. Eur J Endocrinol.

[34] Cosma M, Swiglo BA, Flynn DN, Kurtz DM, Labella ML, Mullan RJ (2008). Clinical review: Insulin sensitizers for the treatment of hirsutism: a systematic review and metaanalyses of randomized controlled trials. J Clin Endocrinol Metab.

[35] Barnes RB (1997). Diagnosis and therapy of hyperandrogenism. Baillieres Clin Obstet Gynaecol.

[36] *Leuprolide [package insert]*. North Chicago, IL: Abbott Laboratories; 2013.

